# Transplantation of human adipose-derived stem cells overexpressing LIF/IFN-β promotes recovery in experimental autoimmune encephalomyelitis (EAE)

**DOI:** 10.1038/s41598-022-21850-9

**Published:** 2022-10-25

**Authors:** Mahdieh Yousefi, Abolghasem Nabipour, Mazdak Ganjalikhani Hakemi, Mehnoosh Ashja-Arvan, Noushin Amirpour, Hossein Salehi

**Affiliations:** 1 Department of Basic Sciences, School of Veterinary Medicine, Ferdowsi University of Mashhad, Mashhad, 91779-48974 Iran; 2 Department of Immunology, School of Medicine, Isfahan University of Medical Sciences, Isfahan, Iran; 3 Department of Anatomical Sciences, School of Medicine, Isfahan University of Medical Sciences, Isfahan, Iran

**Keywords:** Immunology, Neuroscience, Stem cells

## Abstract

Multiple Sclerosis (MS) is the most common demyelinating disease with inflammatory demyelination in the central nerve system. Besides the defect in the myelin repair process, the balance change in inflammatory and anti- inflammatory cytokines is one of the most significant factors in MS pathogenesis. This study aimed at evaluating the effects of co-overexpressing beta interferon (IFN-β) and Leukemia inhibitory factor (LIF) in human adipose-derived stem cells (IFN-β/LIF-hADSCs) on the experimental autoimmune encephalomyelitis (EAE). 12 days after the induction of EAE on female mice C57Bl/6 with MOG35-55 and the emergence of primary clinical signs, the IFN-β/LIF-hADSCs were injected into the mice tail vein of the EAE mice. The mice were sacrificed after 32 days and the spinal cords of the experimental groups were dissected out for the histopathologic and real-time RT-PCR studies. Here, we showed that the clinical scores and infiltration of mononuclear cells of treated mice with IFN-β/LIF-hADSCs were decreased significantly. Demyelination and the number of Olig2^+^ and MBP^+^ cells were significantly increased in the test (IFN-β/LIF-hADSCs) group. The findings revealed that the pattern of inflammatory and anti- inflammatory cytokines gene expression in the IFN-β/LIF-hADSCs group was reversed compared to the control group. Overexpression of LIF as a neurotrophic and IFN-β as an anti-inflammatory cytokine in hADSCs increases the immunomodulatory effect of hADSCs reduces the extent of demyelination, improves the number of Olig2^+^ cells, and also increases the amount of MBP protein which can increase the production of myelin in EAE model. This, besides hADSCs capacity for proliferation and differentiation, might enhance the treatment efficacy and provide a promising candidate for stem cell-based gene therapy of MS therapy in the future.

## Introduction

Multiple sclerosis (MS) is a kind of chronic inflammatory autoimmune disease of the central nervous system (CNS). In MS for unknown reasons, the immune system itself destroys the myelin of neural fibers. The spread and progress of the disease are different and vary from slow to fast-paced disabling progress. Every year thousands of people are diagnosed with MS. The common incidence age is 24–30 but it has been seen from 15 to 50 as well. Women are twice as susceptible to this disease as men^1^.

Different mechanisms like molecular mimicry, release of sequestered antigens, imbalanced Th1/Th2 responses, and nature of antigen-supplying cells contribute to the MS disease^1^. The mouse model of MS called experimental autoimmune encephalomyelitis (EAE), is the most common model for studying the demyelination and inflammatory responses in MS^2^.

In several studies, mesenchymal stem cells (MSCs) have been used for the treatment of autoimmune diseases like MS because of their immunomodulatory effects^3^. MSCs can cure EAE by repressing T cells responses like Th1 and Th17 cell^4,5^. Also, these cells can shift the immune responses toward Th2 and regulatory T cells (Tregs)^6^. Gene therapy by mesenchymal stem cells is recommended for delivery to injured areas by homing and cell division in those areas to improve their therapeutic ability^7,8^. Among mesenchymal stem cells, adipose-derived stem cells (ADSCs) have received more attention due to their high supply, easy access, and sufficient amounts of stem cells in their tissue^9^.

IFN-β, as an interferon type I, owns immunomodulatory effects that can be helpful in the treatment of inflammatory diseases such as relapsing–remitting multiple sclerosis (RRMS). It has been claimed that this cytokine can decrease the relapse of the disease up to 50%, but the main problem is that this medication is not efficacious for 10–50% of the patients^10,11^. During EAE, IFN-β prevents the immigration of inflammatory cells to CNS and also reduces the expression of MHC-II molecules on the surfaces of immune cells, and hence, inhibitsTcell proliferation and finally, cause cytokine environment to shift toward an anti-inflammatory situation^12–14^.

Leukemia inhibitory factor (LIF) is a 180 amino acids length protein that is found in many cells, including fibroblasts, activated T cells, macrophages, chondrocytes, bone marrow stromal cells, mesenchymal stem cells, endothelial cells, astrocytes, and tumor cells^15,16^. The studies on mice without LIF or mice with brain lesions under-treatment with LIF exogenous showed the importance of LIF in different levels of neurogenesis and repair of spinal cord and brain lesions. Also, the effects of external LIF on the reproduction and multiplication of oligodendrocyte progenitor cells and myelin production enhancement have been proved^17,18^. LIF also has been shown to promote OPC differentiation and survival^19,20^. Therefore, we aimed to use these immunomodulatory cytokines (IFN-β and LIF) in human ADSCs which were genetically engineered as a delivery vehicle for continuously expressing IFN-β and LIF in lesion sites of EAE mice.

## Materials and methods

### Isolation and culture of hADSCs

Adipose tissue was obtained from female donors aged 20 to 40 years in Al-Zahra Hospital of Isfahan with informed personal consent and all cell culture experiments involving human ADSCs were approved by the Ethics-Committee of Ferdowsi University of Mashhad (IR.UM.REC.1400.088) and carried out by the approved guidelines. The samples were certified by the surgery room in a sterile container and brought to the laboratory on ice. Then, the following procedure was carried out.

To remove impurities and blood, the adipose tissue was washed several times with phosphate buffer saline (PBS) containing 1% penicillin /streptomycin. After chopping into smaller pieces, the samples were digested with 0.075% collagenase type I (Sigma Aldrich, Germany) prepared in PBS for 30 min at 37 °C.

After 30 min, DMEM with 10% FBS was added to the suspension (1:1 ratio) to neutralize the enzyme. The sample tube was centrifuged for 5 min at 1500 rpm and a stromal vascular fraction (SVF) were cultured in 75 cm2 flasks with DMEM,10%FBS and 1% penicillin/streptomycin solution (in a 37° C wet incubator with 5% CO_2_). 4–5 days after the cell culture, the confluence of adherent cells was 80% (passage 0). For passage, cells were separated from the flask using 0.25% trypsin / 0.02% EDTA (Gibco) and transduced to new flasks (1: 3 ratio)^21^.

### Construction and production lent viral vector and hADSCs transduction

In our previous work^22^ lent viral vector based on HIV-1 that green fluorescent protein (GFP) and IFNβ, human LIF (PCDH 513B 1) gene encoders, as well as vectors (PS PAX2.2), were used as packaging vector (pMD2G) and as Envelope vector. DNA plasmids for new-combined vectors, packing vector, envelope vector, and transfer vectors, were jointly transfected in the HEK293 cell line.

Transduced hADSCs for GFP expression were examined by fluorescence microscopy.

Transduced hADSCs were introduced with vectors containing puromycin resistance genes and GFP genes but no IFNβ and LIF genes as control cells (GFP hADSCs).

### Transduced hADSCs characterization

To confirm the mesenchymal nature of transduced hADSCs, flow-cytometry method was carried out for the analysis of specific surface markers expression (CD markers), and multilineage differentiation capacity was assessed by induction into adipocytes and osteocytes. To investigate the phenotype, the cells from passages 3–5 were incubated by non-human CD45 PerCP, CD73 FITC, and CD105 PE antibodies (all from Bio Legend, San Diego, California, USA) at 4° C for 30 min and then, the panel of surface markers was identified by flow cytometry (Becton–Dickinson, San Jose, CA). Each tube was evaluated 10,000 times and all the data were analyzed by Flowjo software (Tree Star Inc., USA).

To determine the differentiation potential of transduced hADSCs into adipocytes, 1 × 10^3^ cells/cm^2^ of stem cells were cultured in 6-well plates. The culture medium was then switched to a lipid differentiation medium (ready to use) (AdipoPlus, BI 1101). The differentiation medium was refreshed every 3 days. After 21 days, the cells were fixed in PFA 4% and incubated in 0.5% Oil Red O solution in isopropanol (Sigma) for 15 min at room temperature. For osteogenic differentiation, the cells were cultured in the osteogenic induction medium containing ascorbic acid (50 μg/ml), sodium glycerophosphate (10 mm), and dexamethasone (10 mm) (Sigma Aldrich). After 4 weeks, the plates were washed with PBS, fixed with 4% PFA, and then mineralization was confirmed by Alizarin red S staining (2%, pH = 4.1–4.3)^23^.

### EAE induction and treatment protocols

C57Bl/6 female mice (6–8 weeks old) were bought from the Royan Institue of Isfahan. The mice were kept in a controlled condition (Temperature 22–26 °C, humidity 30 40%, natural dry diet) approved by the Animal Ethics Committee for the University of Isfahan. Mice were immunized with 200 µl of myelin oligodendrocyte glycoprotein peptide (MOG35-55, ProSpec) (400 µg)/ Complete Freund's adjuvant (CFA, Sigma) (0.5 mg) emulsion (1:1 vol ratio) which was subcutaneously administered into two sites of each hind flank. Mice were given a total dose of 500 ng of Bordetella pertussis toxin (Sigma) on days 0 and 2 after immunization^24^ (all injections were given through the tail vein) and then randomly divided into the following groups (5 subjects).

1. Control (healthy group), 2. PBS (EAE group), 3. hADSCs (Un-transduced), 4. GFP- hADSCs, and 5. IFN-β/LIF-hADSCs.

On day 12 of post-immunization (DPI), early signs of motor impairment were observed, according to previous studies, the onset of clinical symptoms is due to the inflammatory phase in the EAE model. Therefore, 12DPI was selected for the day of treatment. Befor injection, un-transduced hADSC (passage 3–5) were labeled with PKH26 (Sigma) according to the manufacturer's protocol. For cell transplantation, 1 × 10^6^ cells from each non-transduced and transduced hADSCs were suspended in 100 μl PBS on day 12DPI and injected intravenously into each mouse. For PBS group100µL, PBS was injected intravenously into each mouse on day 12 DPI. Body weight and clinical signs of all mice up to 32 DPI were recorded daily. Clinical signs were scored with: 0, without clinical signs; 1, loss of some tail tonic; 2, complete loss of tail tonicity; 3, loose tail and abnormal gait; 4, hind limb paralysis; 5, paralysis of the hind limbs with hind body passivity; 6, hind- and forelimb paralysis; and 7, death^24^.

### Histological assessment

The spinal cord of mice was quickly dissected after transcranial perfusion (initially with PBS, and then with 4% paraformaldehyde (PFA) mixed with PBS [pH 7.4])) at the time of killing (32 DPI). Tissues were fixed with neutral formalin 10%, embedded in paraffin and serial 5 μm cross-sections were prepared. To investigate the rate of spinal cord inflammation and myelin loss, three sections of each spinal cord area (cervical, thoracic, and lumbar) were stained with hematoxylin and eosin (HE) and three sections with Luxor Fast Blue (LFB) for each animal. The sections were analyzed by a blinded experimenter for the experimental groups. The inflammation score was determined as follows. 0, without inflammation; 1, scattered inflammatory cells; 2, infiltration of inflammatory cells around blood vessels; 3, significant vascular cuff with expansion in adjacent parenchyma^25^. The percentage of the demyelinating area to the whole spinal white matter area was calculated using NIH Image software (http://rsb.info.nih.gov/ij). The following formula is used for the percentage of demyelination amount^26^:

The amount of demyelination (%) = (demyelination zone in white matter)/(total white matter area) × 100%

### Immunofluorescence staining

The immunofluorescence was used to detect the expression of oligodendrocyte markers (Olig2 and Myelin Basic Protein [MBP]). Briefly, after re-dewatering the slides in PBS, permeability was performed by 0.1% Triton X 100 for 1 h at room temperature. Then, the slides were blocked with 3% bovine serum albumin in PBS for 30 min. The sections were incubated with primary antibodies such as Rabbit polyclonal Anti-Olig2 (1:1000) (ab136253, Abeam, Cambridge City, MA, USA) and goat polyclonal Anti-MBP (1:200) (sc-13914; Santa Cruz Biotechnology, Inc., Santa Cruz), CA) overnight at 4 °C in a humidified chamber. After washing 3 times with PBS at room temperature, the sections were incubated with highly cross-adsorbed conjugated secondary antibodies, such as Goat Anti-Rabbit FITC (1:1000) (ab6717; Abeam, Cambridge City, MA, USA), Goat Anti-Rabbit Phycoerythrin (1: 1000) (ab72465; Abeam, Cambridge City, MA, USA), Donkey Anti-Goat IgG FITC (1: 1000) (ab6881; Abeam, Cambridge City, MA, USA), Donkey Anti-Goat Phycoerythrin (1: 1000) (ab7004; Abeam, Cambridge City, MA, USA) for an hour at room temperature. Finally, after washing 3 times with PBS, the tissues were incubated for 5 min with diamidino phenyl indole (DAPI) and were seen with a fluorescence microscope (Olympus, BX51, Japan). Quantitative analysis of positive cells in three vision fields per Sect. (200 / 400X magnification) was performed by Image and the numbers of Olig2 and MBP-labeled cells were counted. Moreover, three mice from each group were randomly selected^21^.

### The analysis of gene expression in CNS

To determine the expression of inflammatory and anti-inflammatory cytokines, the spinal cord was removed from the experimental groups at 32 DPI. Complete RNA was isolated from tissue using Bio FACT ™ Total RNA Prep Kit and RNA concentration was determined by NanoDrop spectrophotometer. cDNA synthesis was obtained from 1 μg of total RNA with cDNA reverse transcription kit (Bio FACT). The purity of the obtained a cDNA was controlled by NanoDrop. To detect the expression of five genes like interleukin 17a (IL-17a), tumor necrosis factor α (TNF α), interferon γ (IFN γ) (as inflammatory cytokines) and IL 4 and IL 10 (anti-inflammatory cytokines), qtr-PCR was performed in duplicate using SYBR® Green PCR Master Mix (Bio FACT) containing 5 μl SYBR Green, 1 μl of cDNA, 0.25 μl of primer (forward and reverse mixture) and 3.75 μl of nuclease-free water. Β-actin was determined as a control gene for this study. All genes were calculated using the relative quantification method (2^−∆∆CT^) according to the standard protocol and mRNA expression was considered equal to the control for analysis^27^. The specific primers of the above genes were designed in our laboratory by Allele ID software, version 7.0, and their sequences are listed in Table 1.

**Table 1:** The sequences of the applied primers in qtr.-PCR.

Primer name	Sequence (5’ → 3’)	Accession number	Size
IL-4	Forward : 5’-AGTTGTCATCCTGCTCTTCTT-3’	NM_021283.2	169
	Reverse : 5’- TGTGGTGTTCTTCGTTGCT-3’		
IL-10	Forward : 5’- GCTATGCTGCCTGCTCTT-3’	NM_010548.2	222
	Reverse : 5’- CAACCCAAGTAACCCTTAAAGT-3’		
IL-17a	Forward : 5’- GACTCTCCACCGCAATGA-3’	NM_010552.3	204
	Reverse : 5’- ACACCCACCAGCATCTTC-3’		
IFN-γ	Forward : 5’- AAAGAGATAATCTGGCTCTGC-3’	NM_008337.4	229
	Reverse : 5’- GCTCTGAGACAATGAACGCT-3’		
TNF-α	Forward : 5’- GTGGAACTGGCAGAAGAG-3’	NM_013693.3 M	228
	Reverse : 5’- TTGAGAAGATGATCTGAGTGT-3’		
β -actin	Forward : 5’- GGCTGTATTCCCCTCCATCG-3’	NM_007393.5 M	154
	Reverse : 5’- CCAGTTGGTAACAATGCCATGT-3’		

### Statistical analysis

Data were reported as Mean ± SEM and analyzed using SPSS version 23 (SPSS Inc., Chicago, IL, USA) and Graph Pad Prism software (version 8.01). Differences in body weight variables were assessed using a repeated measures test followed by a comparison of Bonferroni and other variables were analyzed using one-way analysis of variance (ANOVA) followed by the Tukey post hoc test.

The statistical analyses of clinical and histopathological scores were assessed using repeated non-parametric Kruskal–Wallis test. The scores represent ordinal (ranked) data.

*P* value < 0.05 was considered as the minimum level of significant difference.

### Ethics declarations

Ethical approval for this study was approved Ethics Committee of Ferdowsi University of Mashhad (IR.UM.REC.1400.088). All experiments were performed by relevant guidelines and regulations.

This study followed the recommendations in the ARRIVE guidelines.

## Results

### hADSCs transduction and selection

As shown in our previous work, hADSCs were transduced with a lentiviral vector expressing IFN‐β and LIF. Briefly, after assembly of lentiviral vector (pCDH‐513B‐1, ps‐PAX2.2, and PMD2.G) in Hek293T cells, the supernatant was collected and the virus was concentrated. Afterward, hADSCs in the third passage were transduced with the lentiviral vector expressing IFN‐β and LIF, and then, transduced cells were selected with puromycin and evaluated with fluorescence microscopy and flow cytometry analysis. Our data revealed high‐efficiency transduction with the highest expression rates on day 75 after transduction which were 70 pg/ml for IFN‐β and 77.9 pg/ml for LIF in comparison with 25.60 pg/ml and 27.63 pg/ml, respectively, in untransduced cells.

### Fluorescence study of IFN-β/LIF-hADSCs

**Figure 1:**
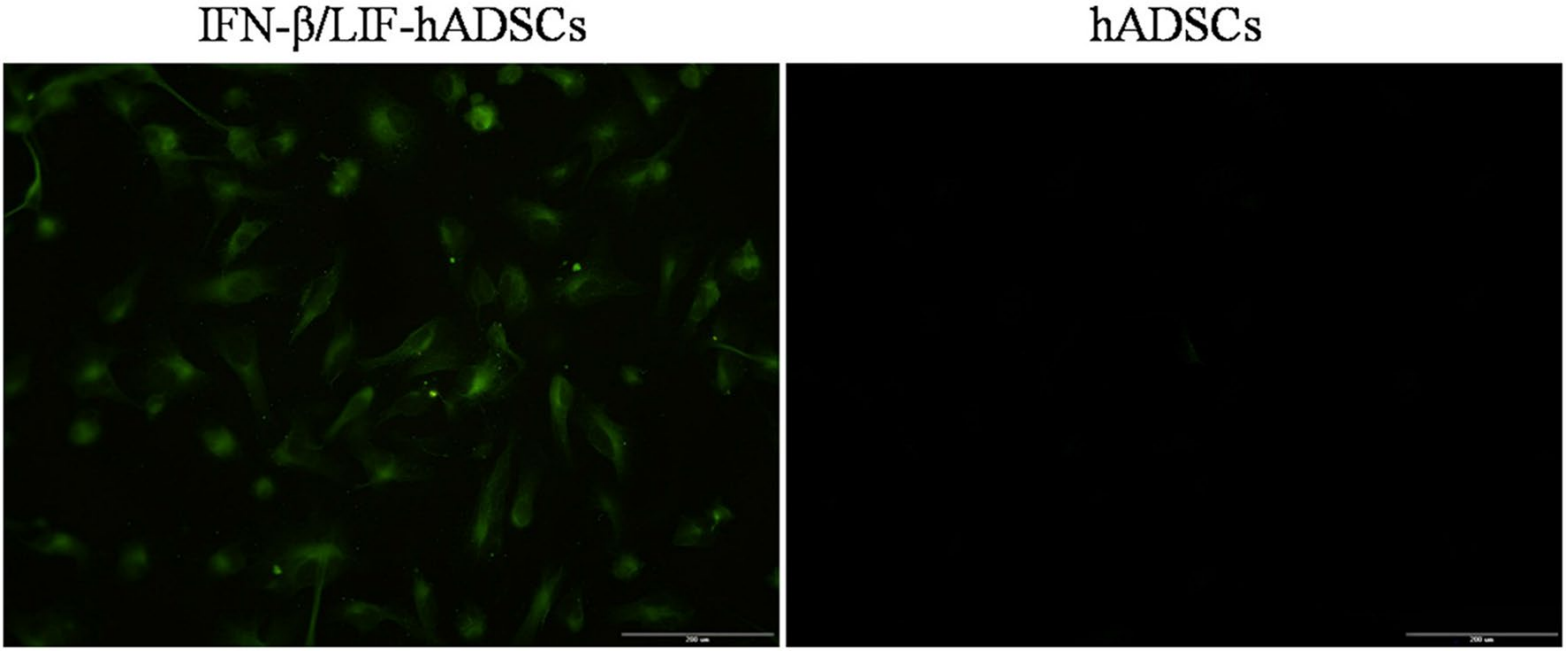
Fluorescence microscope examination of overexpressed IFN-β and LIF in hADSCs that co-express GFP from the LV/IFN-β/LIF/GFP vector.

Transduced hADSCs for GFP expression were examined by fluorescence microscopy. A high level of GFP signal was detected in the cytoplasm of transduced hADSCs, indicating that our lent viral vector was able to deliver the exogenous IFN‐β and LIF genes into the hADSCs and permit expression in the nuclei (Fig. 1). High GFP expression showed a high rate of transduction.

### The characteristics of transduced hADSCs

**Figure 2:**
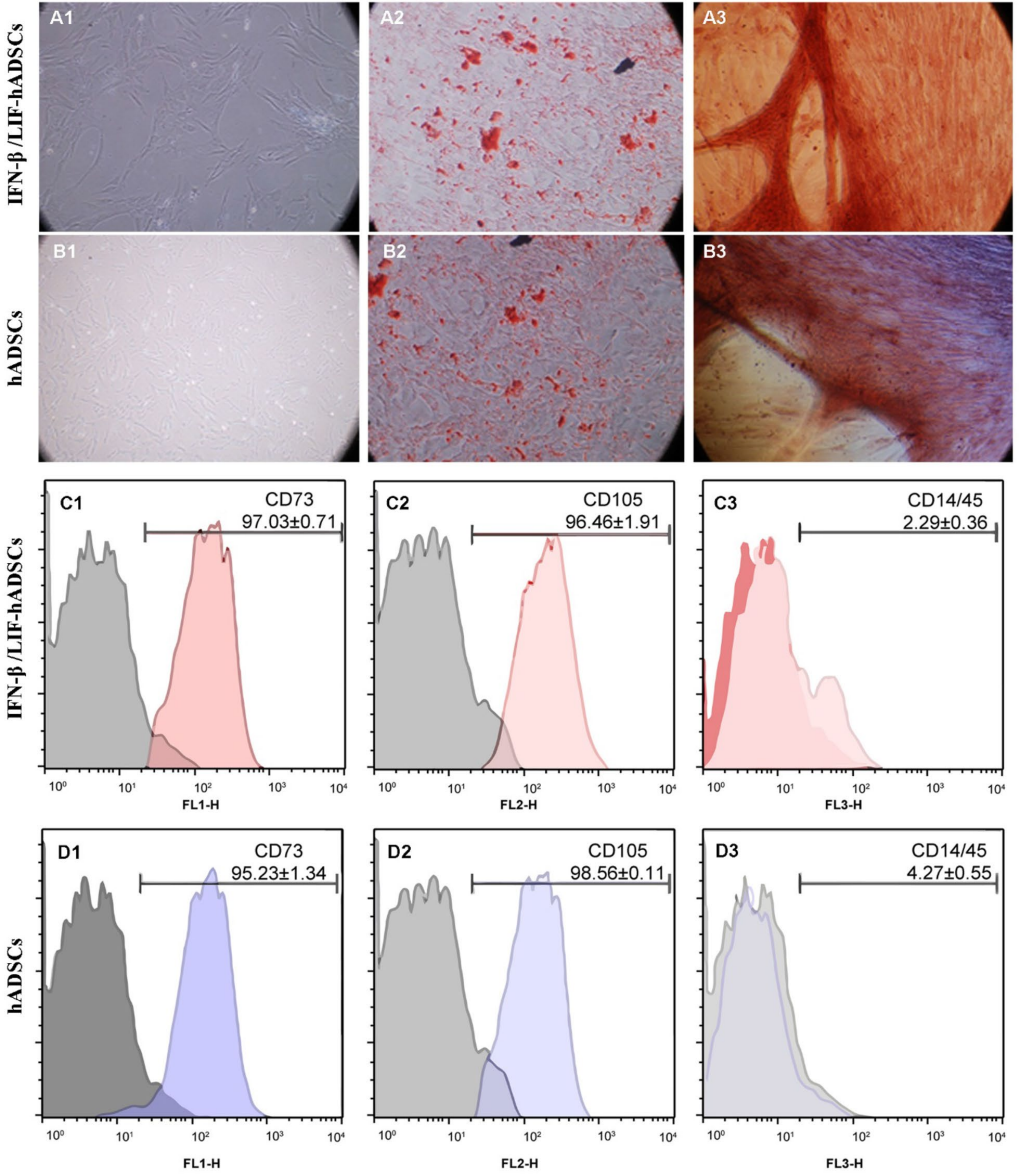
Differentiation potential and cell surface characterization of transduced and un-transduced hADSCs. Phase contrast microscopy (**A1** and **B1**) images show fibroblast-like morphology of transduced hADSCs. For abiogenic and osteogenic differentiation, transduced hADSCs were stained with Oil red (**B2**, **A2**) and Alizarin-Red (**A3**, **B3**), respectively. Scale bar, 100 µm. For the immunophenotypic profile of the transduced hADSCs, the cells were stained with selected CD marker antibodies (positive for CD73 and CD105, negative for CD14/45) (**C**, **D**). All cells were selected from the 3rd to 5th passages.

Three days after culturing, the primary cells were fully elongated and became spindle-shaped, and fibroblast-like cells. In addition, after transducing IFN-β, LIF, and GFP into hADSCs, no significant differences in cell size, proliferation, morphology, and potential were observed between overexpressed and un-transduced stem cells (Fig. 2A1, B1). Oil red staining showed a significant number of fat droplets 21 days after abiogenic differentiation of transduced hADSCs (Fig. 2A2, B2). In addition, Alizarin-Red staining showed a large amount of calcium deposition (Fig. 2A3, B3). These observations showed the differentiation capacity of transduced hADSCs toward adipose and bone cells, respectively. Immunophenotypic analysis showed that both cell groups (IFN-β/LIF-hADSCs and hADSCs) were positive for mesenchymal stems cell markers such as CD73 and CD105 but negative for CD14/45 (hematopoietic stem cell markers) (Fig. 2C, D). These data confirmed that the selected stem cells were mesenchymal stem cells and the vector transducing did not change the mesenchymal properties of the transduced hADSCs.

### IFN-β/LIF-hADSCs improved the clinical score of EAE mice

**Figure 3:**
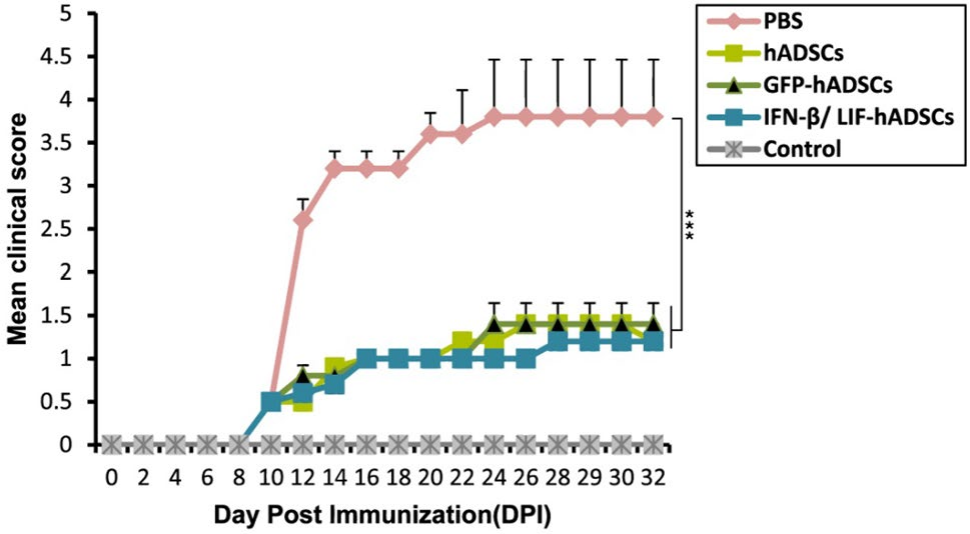
hADSCs overexpressing IFN-β and LIF reduced the severity of EAE disease in immunized mice. Clinical scores were monitored daily in all five groups for up to 32 days after immunization. Generally, there were significant differences in the mean clinical scores among the three treatment groups and the PBS group [H(3) = 110.456, *p* = 0.000]. Results were shown as mean ± SEM. *P < 0.05, **P < 0.01, ***P < 0.001. Nonparametric Kruskal–Wallis tests were used to compare the clinical scores among the groups.

To investigate the effect of un-transduced and transduced hADSCs in the treatment of EAE mice, an intravenous injection of 10^6^ cells was performed at the onset of the inflammatory phase of the disease (on day 12 DPI) when the first symptoms (loss of tail tonicity) were seen. Up to 32 DPI, Mice clinical scores were recorded. Overall, the clinical score decreased in the three treatment groups compared to the untreated EAE group. Maximum clinical scores (mean ± SEM) for PBS, GFP, hADSCs, and IFN-β/LIF-hADSCs groups were 3.8 ± 0.69, 1.4 ± 0.15, 1.4 ± 0.08, and 1.2 ± 0.10, respectively, which in IFN-β/LIF-hADSCs group was significantly lower than the other three treatment groups (*P* < 0.001). On the last day after immunization (32DPI), the clinical score means of treated mice decreased significantly compared with untreated EAE (PBS), (*P* < 0.001) (Fig. 3).

### IFN-β/LIF-hADSCs limited the weight loss in the EAE mice

Two days after MOG vaccination, a weight loss was observed in all mice, our information within 32 days showed that the body weight in treatment groups was significantly higher than the PBS group. Meanwhile, the body weight means in the PBS group was significantly decreased (DPI32).

The mean weight of mice in Control (20.39 ± 0.19), PBS (17.30 ± 1.16), IFN-β/LIF-hADSCs (17.30 ± 0.27), GFP (18.34 ± 0.38) and hADSCs (17.30 ± 0.58) groups had not significantly changed in DPI12.

**Figure 4:**
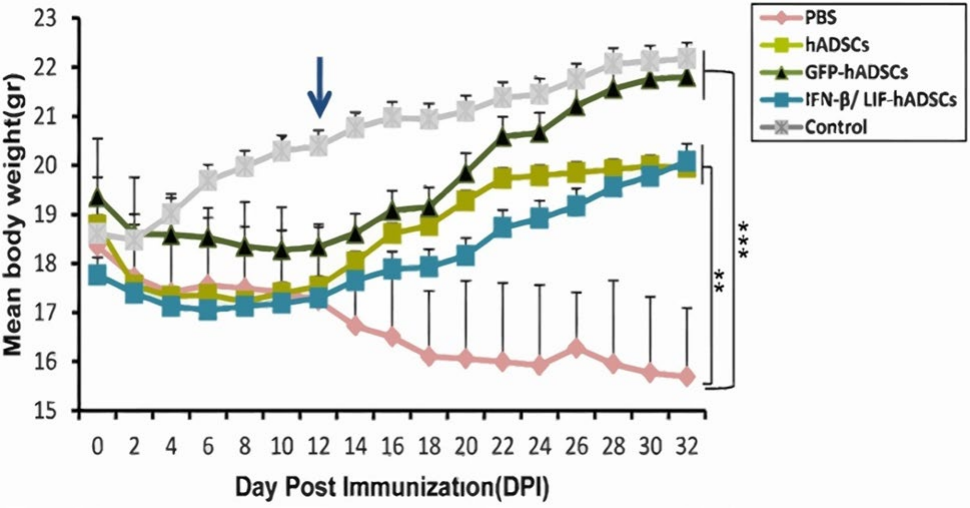
hADSCs overexpressing IFN-β and LIF reduced the weight loss in immunized mice. The body weight of all five groups was monitored daily for up to 32 days after immunization. In general, the mean body weight of the three treatment groups differed significantly compared with PBS mice (F9,14 = 12.951, *P* = 0.00003). Body weight changes in the three treatment groups compared to the healthy group were not significant. Results were shown as mean ± SEM. *P < 0.05, **P < 0.01, ***P < 0.001.

In DPI32, the mean weight of Control (22.18 ± 0.19), PBS (15.69 ± 1.18),

IFN-β/LIF-hADSCs (20.08 ± 0.18), GFP (21.81 ± 0.17), and hADSCs (20.08 ± 0.02) groups indicated a significant improvement in body weight in the three treatment groups. Generally, there was no significant difference between the treatment groups and the healthy group while there was a significantly better recovery from weight loss between the treatment groups and the PBS group (*P* < 0.001) (Fig. 4).

### hADSCs overexpressing IFN-β and LIF diminished the penetration of mononuclear cells and reduced the resulting demyelination in EAE mice

**Figure 5:**
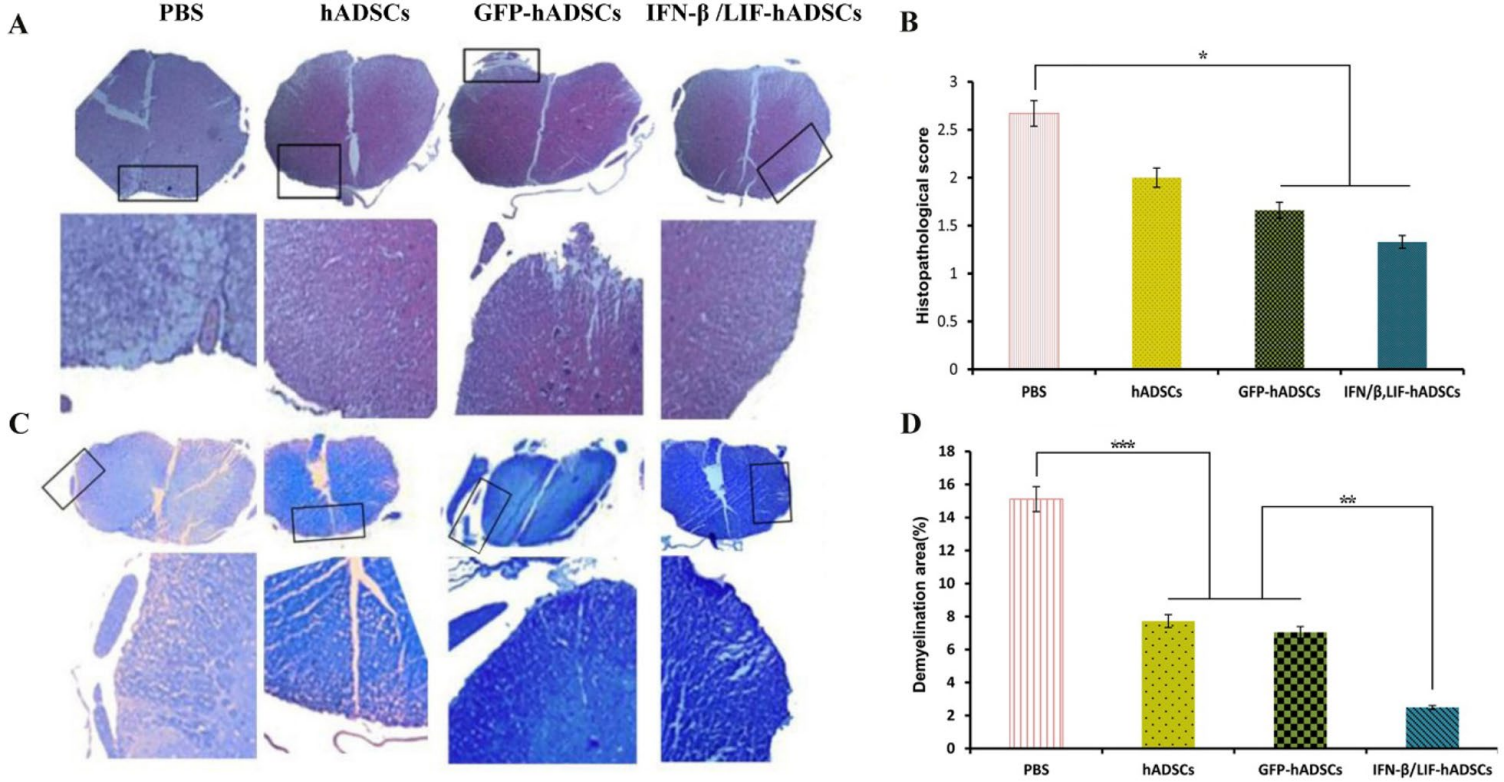
Histopathological cross-section of the spinal cord in different groups. Histopathological staining of spinal cord sections of animals 32 days after immunization for cell infiltration (H&E) and demyelination (LFB). A and B, respectively; 40X magnification for original images and 200X magnification for inserts. Quantitative cell diffusion (H&E) results were analyzed by Kruskal–Wallis and demyelination (LFB) results were analyzed by one-way ANOVA. B, (*P* = **.**037) D F3,8 = 63.038, *P* = 0.000007). (mean ± SEM, *P < 0.05, **P < 0.01, ***P < 0.001).

To investigate the effects of cell transplantation on histopathological changes (the penetration of immune cells and demyelination) in the EAE mice, H&E and LFB staining were performed. H&E staining showed the penetration score of mononuclear cells (inflammation) in the spinal cord of the groups. By the results of the clinical score, analysis of H&E images showed that the inflammation score in all treatment groups was significantly reduced compared to the PBS group (*P* < 0.001) (Fig. 5A, B). Furthermore, in IFN-β/LIF-hADSCs and GFP groups, the inflammatory score was significantly lower than hADSCs groups (respectively, 1.33 ± 0.34, 2 ± 0.58, and 1.66 ± 0.36) (*P* < 0.001) (Fig. 5B). Also, a significant difference has existed between two treatment groups and PBS group (PBS:2.67 ± 0.18). LFB staining was used to evaluate demyelination in experimental groups. Quantitative analysis of LFB images showed a focal decrease in the percentage of demyelinated areas in the PBS group. In contrast, as was shown in the image (Fig. 5C, D), treatment groups showed a notable decrease in demyelination area compared to the PBS group (PBS:14.90 ± 0.95) GFP: 7.04 ± 1.19, hADSCs: 7.72 ± 0.57 and IFN-β/LIF-hADSCs: 2.49 ± 0.27) (*P* < 0.001). Also, there was a significant difference between the IFN-β/LIF-hADSCs group and the two other treatment groups (*P* < 0.01). Interestingly, the lowest amount of demyelination was seen in the IFN-β/LIF-hADSCs group (*P* < 0.001).

**Figure 6:**
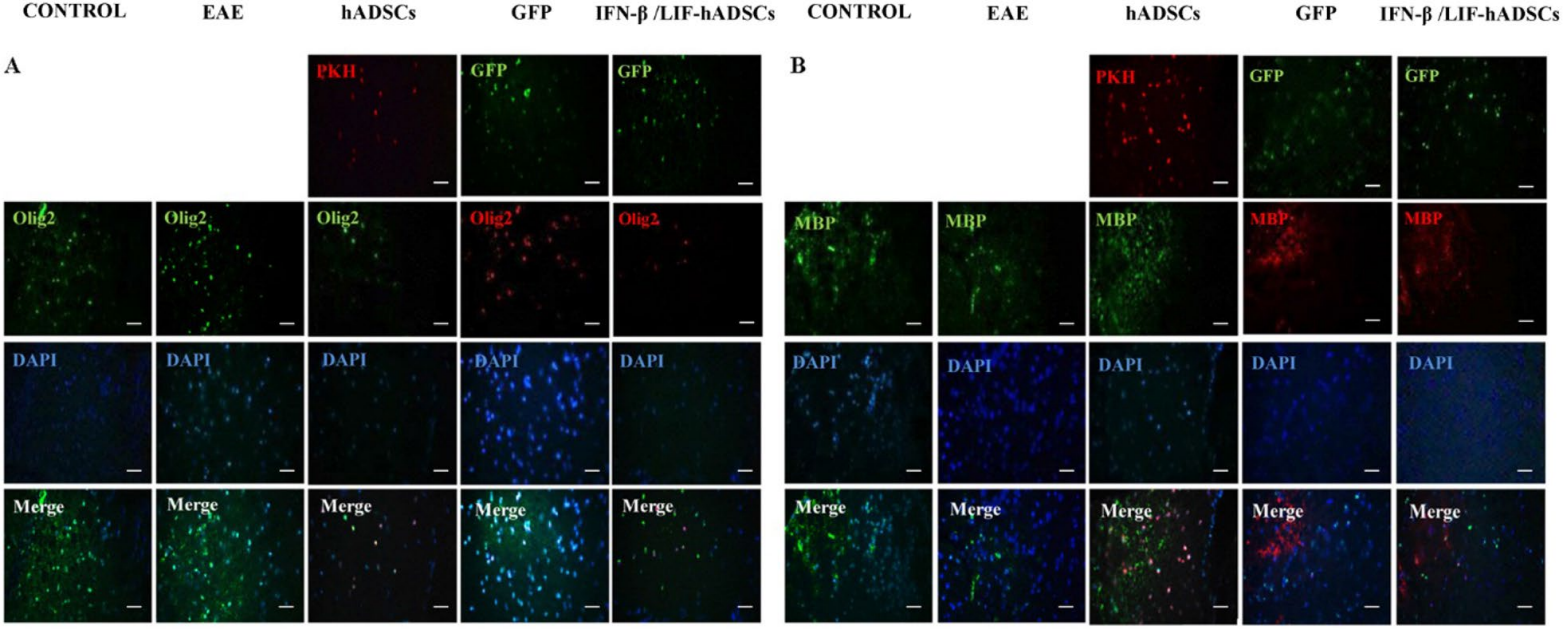
Fluoromicrographs of oligodendrocyte markers in hADCs. Immunofluorescence staining of selected spinal cord slides of five groups with antibodies (A, Olig2, and B, MBP).

**Figure 7:**
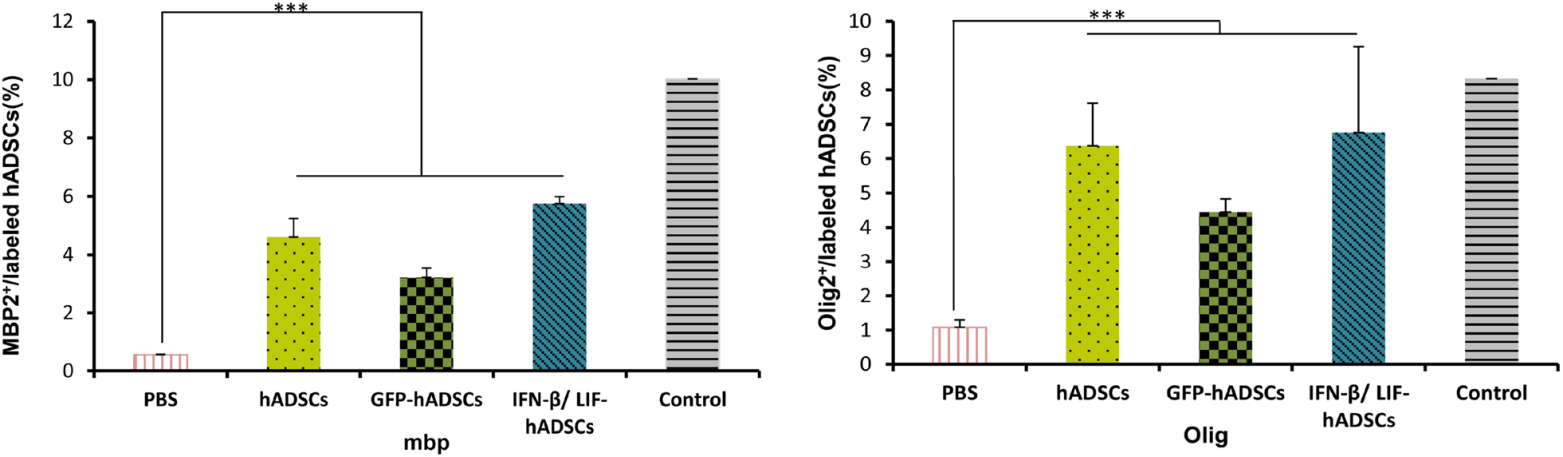
The quantification of Olig2 and MBP positive cells in immunostained spinal cord sections (A, B, respectively). Results are presented as mean ± SEM. Statistical analysis was performed through one-way ANOVA. *P < 0.05, **P < 0.01, ***P < 0.001, *n* = 3). MBP: (F4,8 = 21.809, *P* = 0.00023), Olig2. (F4,8 = 25.620, *P* = 0.000129).

Immunofluorescence staining was used to evaluate immature and mature oligodendrocytes obtained from transplanted hADSCs in spinal cord lesions (Olig2 and MBP, respectively) (Fig. 6). The results showed that the Olig2^+^ cell percentage was 6.37 ± 1.23 in hADSCs group, 4.44 ± 0.38 in GFP group, 6.75 ± 2.49 in IFN-β/LIF-hADSCs, 8.3302 ± 0.25 in the control group and 1.08 ± 0.21in the PBS group, and the percentage of MBP^+^ cell was 4.61 ± 0.62 in the hADSCs group, 3.20 ± 0.32 in the GFP group and 5.75 ± 0.23 in the IFN-β/LIF-hADSCs group, 10.01 ± 2.58 in the control group and 0.55 ± 0.001 in the PBS group. These findings indicated that the expression of human Olig2 protein and MBP in treated mice showed a significant increase compared with the PBS and EAE groups (*P* < 0.001) but did not show a significant difference with the healthy group (Respectively, *P* < 0.001 and *P* < 0.05) (Fig. 7).

### hADSCs overexpressing IFN-β and LIF adjusted the balance between pro- and anti-inflammatory cytokines

**Figure 8:**
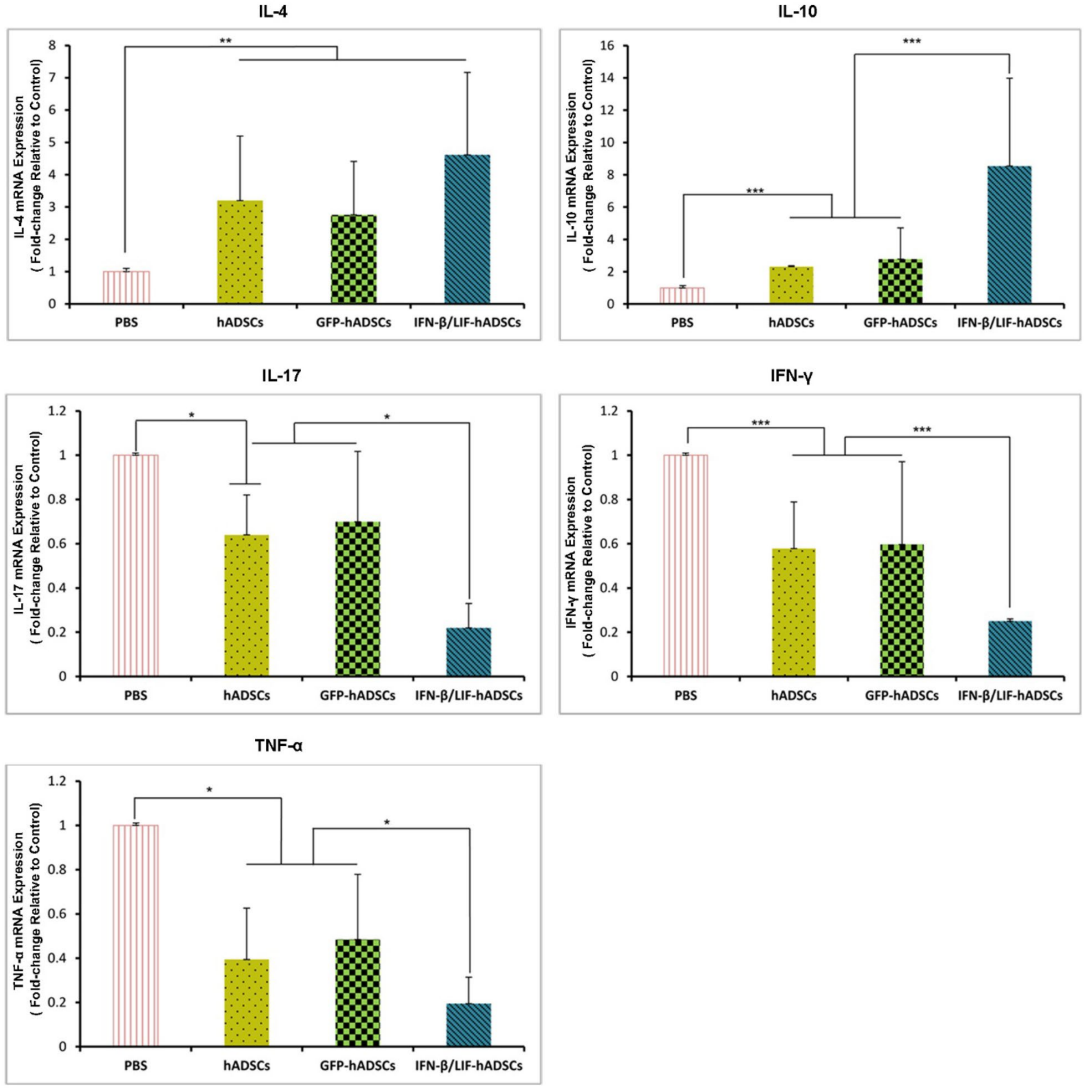
Cellular treatment affected the differential expression of pro/anti-inflammatory cytokine genes in CNS experimental groups. Quantitative RT- PCR data from spinal cord homogenates showed significant downregulation of pro-inflammatory cytokines (IL- 17, IFN -γ , and TNF -α) and upregulation of anti-inflammatory cytokines (IL-4 and IL-10) in the treated groups compared to an untreated group that was very prominent in IFN-β/LIF-hADSCs group. (The results are shown as mean ± SEM of three animals, *P < 0.05, **P < 0.01, ***P < 0.001 (One-way ANOVA).IL-4:(F3,8 = 17.761, *P* = 0.001), IL-10:(F3,8 = 33.937, *P* = 0.000) IL-17:(F3,8 = 4.250, P = 0.045), IFN –γ:(F3,8 = 197.041, *P* = 0.000), TNF –α:(F3,8 = 4.570, *P* = 0.038).

To evaluate the immune modulating effects of hADSCs overexpressing IFN-β and LIF on the EAE mouse model, we assessed the expression of inflammatory and anti-inflammatory cytokines in experimental groups by qtr.-PCR. The results showed the downregulation of IL-17, IFN-γ, and TNF-α (as inflammatory cytokines) in treated groups compared to the PBS group. Among treated groups, the lowest expression level of these factors was seen in the IFN-β/LIF-hADSCs group (*P* < 0.001). The results also revealed the upregulation of IL − 10 and IL − 4 (as anti-inflammatory cytokines) in treated groups in comparison to the PBS group. The highest gene expression of anti-inflammatory cytokines belonged to the IFN-β/LIF-hADSCs group and a significant upregulation of IL-10 was detected in the IFN-β/LIF-hADSCs group compared to the other two treatment groups (*P* < 0.001). In our study, IL- 4 expression was indeed significantly increased in all treated groups compared to the PBS group (*P* < 0.001) (Fig. 8).

## Discussion

This study aims to investigate the functional quality of hADSCs with the overexpression of IFN-β and LIF and whether these cells have the potential to be used as a reliable tool for stem cell-based gene therapy.

In the present study, it was shown that neuronal dysfunction, demyelination and inflammation scores in EAE mice were significantly improved by intravenous administration of IFN-β/LIF-hADSCs. Recently, hADSCs-based therapy studies have indicated that the use of these cells in demyelinating diseases like MS is highly promising. The effectiveness of these cells in the treatment of these diseases is owing to easy access, easy culture and spread, and secretion of anti-inflammatory substances and chemokines, which can be used as a suitable cell therapy device to treat inflammatory and autoimmune diseases^28^. Numerous studies have demonstrated that hADSCs cells lead to the development of treatment strategies in the MS mouse model, EAE^6,29^. These findings have resulted in several clinical experiments using these this cells^30^. Some researchers have used hADSCs cells as well as IFN-β to treat MS model in animals^31,32^. Understanding the properties of hADSCs cells may be particularly important in its future therapeutic applications. Although the exact mechanism of migration of mesenchymal stem cells into inflammatory CNS tissue has not yet been determined, it was reported that ADSCs expressing α4β1 integrin have a special ability to migrate and penetrate into nerve tissue^33^. Regarding the migration, some studies revealed that GFP-containing hADSCs used in the EAE model was also found in the inflammatory CNS tissue up to three months after injection^34^. According to these findings, the invocation of hADSCs to the site of inflammation in the EAE model appears reasonable. Given that conventional pharmacological approaches are ineffective to treat MS patients owing to their side effects and continuous therapeutic doses^35^. Mesenchymal stem cells are recommended as a source to regulate the secretion of cytokines, which affect both the innate immune system and the adaptive immune system^36^. Owing to the anti-inflammatory and immunomodulatory properties of hADSCs, they can be a good source for stem cell-based gene therapy in MS disorders^35,37^ MS is a chronic inflammatory disease requiring long-term treatment, and IFN-β is currently used as the first-line treatment for MS^38^. This medication is usually applied at the peak of the disease and has its disadvantages. Since, frequent use of its, which is a protein compound, activates the immune system against it and leads to the production of neutralizing antibodies, thereby reducing the medication effectiveness^39^. Therefore, to reduce the side effects of the long-term use of IFN-β in the treatment of MS, we used IFN-β and LIF gene transduced hADSCs to treat the EAE mouse model. According to our results, the overexpression of IFN-β/LIF-hADSCs can be superior to its protein form.

In the first part of our study, overexpression of IFN-β and LIF by hADSCs was investigated, and ELISA results at 15, 45 and 75 days after stem cell transduction, revealed that the concentration of therapeutic cytokines was significantly higher in the supernatant of transduced hADSCs compared to non-transduced hADSCs^22^. The data indicated that hADSCs could be considered a good transducer for the long-term expression of therapeutic cytokines^40,41^.

Previous studies had contradictory results that the use of IFN-β in a new form had protective or worsening effects. However, this worsening condition was reported in patients whose disease was dominated by Th17 cells, but in patients with the Th1 profile, the use of IFN-β improved the disease^42,43^. Our evidence indicated that the use of IFN-β and LIF improved the condition in the EAE model. Unlike studies using IFN-β as a protein, we used it as a gene inside hADSCs cells, which might provide us the opportunity for long-term expression in the body, thereby reducing the harms and disadvantages of this protein. Furthermore, we observed synergistic effects of these cells owing to the secretory factors which increase the immunomodulation of this treatment process. In addition, according to other confirmed studies, the basic characteristics of hADSCs did not change with genetic modification and were similar to stem cells^35,37^. As mentioned in many studies, one of the most significant features of using these cells to treat MS is related to the suppressive nature of immune responses, particularly the prevention of T cell proliferation, including Th1 and Th17, which are among the most important immune cell disruptors in this disease. Additionally, mesenchymal stem cells can prevent the expression of antigens by antigen-supplying cells; these cells have recently been used to treat graft versus host disease (GvHD) allogeneic ally^38^. In the animal model of EAE, similar to MS, Th1 and Th17 cells activated by the blood–brain barrier (BBB) ​​migrate to the CNS tissue. These subset cells produce inflammatory cytokines leading to neuroinflammation and demyelination^44,45^. Clinical signs have resulted from the CNS demyelination following inflammation in EAE mice^46^. In the second part of our study, we evaluated the effect of IFN-β/LIF-hADSCs on the regulation of immunoregulation and demyelination in the CNS of EAE mice by intravenous injection. The H&E staining results showed the infiltration of abundant mononuclear cells into the white matters of the spinal cord in untreated EAE mice, which was reduced in IFN-β/LIF-hADSCs groups compared to the other two treatment groups of hADSCs. Lymphocytic infiltration is a prominent feature of the central nervous system tissue in EAE^47^. Studies have demonstrated that MS and autoimmune encephalomyelitis are experimental inflammatory diseases associated with lymphocyte infiltration into brain and spinal cord tissues. Cells invading the brain tissue cause inflammatory reactions in the brain tissue, thereby causing myelin destruction. The severity of the lesions depends on the degree of cell infiltration^48,49^. In addition, some studies have reported an increase or decrease in the number of Th17 penetrating CNS with IFN-β prescription. The results of the present study showed that, compared to other groups, the use of IFN-β/LIF-hADSCs reduced lymphocyte infiltration into the brain in mice with EAE. According to a previous study, IFN-β contributes to treatment in at least two cases, first by maintaining the strength of the blood–brain barrier tissue, and second by preventing the migration of lymphocytes to the brain^50^.

Analysis of LFB images showed a significantly less demyelination area in the spinal cords of mice treated with IFN-β/LIF-hADSCs as compared to other experimental groups. In autoimmune diseases, myelin destruction and oligodendrocyte apoptosis have been reported in the EAE model^51^. Therefore, in lesions, re-gathering of oligodendrocytes may play a crucial role in myelin regeneration. LIF as a known pro-remyelination factor could exert a beneficial effect in the circumstance of a toxic oligodendrocytopathy and also could potentially protect oligodendrocytes in the context of MS, especially in active plaques where the blood–brain barrier is acutely disrupted^52^. Additionally, in a previous study, the authors revealed that NG2-targeted LIF-PLGA nanoparticles promote OPC maturation into mature oligodendrocytes invitro and improve CNS remyelination both in terms of the percentage of fibers remyelinated and their thickness (maturity of the sheath)^53^. Moreover, LIF can enhance b-galactosidase expression in oligodendrocytes obtained from mice in which the LacZ gene was driven by the promoter of MBP. This suggested that LIF family members act either directly or indirectly to enhance key myelin gene synthesis^52^. Past data indicated that local expression of LIF has a beneficial effect on CNS lesion development as it significantly reduced the extent of demyelination. Administration of neutralizing anti-LIF antibodies in EAE increases the extent of acute demyelination and doubles the OLG loss already induced by EAE.

Increased oligodendrocyte survival in response to systemic LIF may be mediated through its ability to induce the expression of NT-3, a known survival factor for oligodendrocytes^54^. In other words, IFN-β and LIF with the neuroprotective effects of hADSCs are oligodendrocyte activating agents that improve the number of Olig2^+^ cells as well as increase the amount of MBP, which can increase myelin production.

Olig2 is one of the main factors in the process of reproduction and elimination of OPC development ^55^. In the present study, the expression of human Olig2^+^ and MBP^+^ in the treated groups were significantly increased compared to PBS group and the superior expression was detected in IFN-β/LIF-hADSCs group compared to the other treatment groups. These expressions might be partly related to the transdifferentiation of hADSCs into oligodendrocyte-like cells. However, this transdifferentiation is controversial. Ying et al. and Terada et al. express doubts concerning the occurrence of this differentiation. They co-cultured embryonic stem cells (ES) and adult stem cells to transdifferentiate toward embryonic-like stem cells.The results indicated that adult stem cells fused with the ES cells and took on their properties. Therefore, the ES-like cells arose from the fusion of the neural and embryonic stem cells, rather than from transdifferentiation. But from another side, the transdifferentiation rate has been seen to occur in 7–57% of the neural stem-cell population in co-culture assays whereas cell fusion occurs at the lower frequency of 1 in 10^4^ to 1 in 5 × 10^5^^56–58^. Consequently, all of these in-vivo differentiations were unable to account for only by cell fusion of adult stem cells.

To demonstrate that our therapeutic cells have immunomodulatory properties, we compared proinflammatory cytokines, including IL-17, TNF-α and IFN-γ, and anti-inflammatory cytokines such as IL-4 and IL-10. In addition, our results showed that these cells were well able to increase these anti-inflammatory cytokines and decrease pro-inflammatory cytokines.

IL-17, TNF-α, and IFN-γ are proinflammatory cytokines. These cells are produced by various innate and adaptive immune cells^59,60^. In the EAE model, overexpression of these inflammatory cytokines, along with the auto reaction of The cells was observed^61,62^. Owing to the significance of these cytokines in the pathogen EAE^63^. To demonstrate that hADSCs cells have immunomodulatory properties, we compared pro- inflammatory cytokines, such as IL-17, TNF-α, and IFN-γ, and anti-inflammatory cytokines such as IL -4 and IL -10. Moreover, our results indicated that these cells were perfectly able to increase these anti-inflammatory cytokines. We studied the inflammatory and anti-inflammatory levels of the mRNA expression and the beneficial effects of IFN-β/LIF-hADSCs in inhibiting the expression of pro-inflammatory cytokines in the EAE module, a significant downregulation of pro-inflammatory cytokines is shown in groups treated by hADSCs, in the present study. Recent studies have reported that mesenchymal stem cells derived from the human bone marrow tissue reduce the severity of EAE by inducing immune responses to Th2, reducing IFN-γ and Th17 cells 8 Our results revealed that these cells with IFN-β and LIF had positive effects on the CNS site, at least in terms of infiltration of inflammatory cells and increase of immune suppressor cells in the spinal cord. These cells, similar to those derived from bone marrow tissue, cause modulatory changes in lymphocyte cells and reduce the proliferation and production of inflammatory cytokines in vitro. The present study showed a significant reduction in the number of pro-inflammatory cytokines in hADSC-treated groups. Based on these findings, it appears that IFN-β/LIF-hADSCs can have considerable potential in reducing the penetration of mononuclear cells by suppressing the expression of pro-inflammatory cytokines^64^. The present study showed a significant reduction in the number of pro-inflammatory cytokines in hADSC-treated groups. Based on these findings, it appears that IFN-β/LIF-hADSCs can have considerable potential in reducing the penetration of mononuclear cells by suppressing the expression of pro-inflammatory cytokines. Moreover, the expression of anti-inflammatory cytokines (IL- 10, IL -4) in the experimental groups was detected. High levels of IL-4 and IL-10 in IFN-β/LIF-hADSCs groups could indicate that IFN-β increased the production of anti-inflammatory cytokines and reduced inflammatory cytokines. CNS plays a significant role in regulating the immune system by inhibiting the autoimmune activity of CD4^+^ T cells in inflammatory disorders^50^. TGF-β also plays a key role in the conversion of Treg and Th17 cells to each other. This process requires another cytokine environment. For example, in the case of Th17, cytokine IL- 6 plays a critical role, but TGF-β as an important anti-inflammatory cytokine can play a role in the induction of Treg cells. The interpretation of the results is highly controversial due to the various factors involved in studies related to Treg cells, and there are various reports in this regard so that it cannot be judged only by investigating one factor. Another study demonstrated that stem mesenchymal cells from bone marrow tissue reduced the expression of t-bet and GATA3, and consequently reduced the levels of IFN γ^65^.

An important issue to consider in interpreting the results of this study is the rate of cell injection as well as the time of injection during the disease. As mentioned in many previous experiments, the effectiveness of stem mesenchymal cells in MS with its animal model, EAE, is highly dependent on the amount of injection and pre-disease cell injection as prevention^29,50^. It should be noted that in this study, cell therapy was started when the disease had started, and the mice were clinically at a score of 0.5. In one study by other researchers, the injection was given on day 7 after immunization of the mice, and their results revealed that in the group injected with IFN-β transduced stem mesenchymal cells, the anti-inflammatory cytokine IL 10 was increased, and the inflammatory cytokine IFN γ was decreased^50^.

Overall, the current findings suggest that hADSCs overexpress the effects of IFNβ and LIF on the treatment of autoimmune diseases of the CNS.

## Conclusion

Our experimental approach confirms that overexpression of LIF as a neurotrophic and IFN-β as an anti-inflammatory cytokine in hADSCs increase the immunomodulatory effect of hADSCs and reduce demyelination and improve the number of Olig2^+^ cells and also increase the amount of MBP protein, which can increase the production of myelin in the EAE model. This, besides hADSCs capacity for proliferation and differentiation, might enhance the treatment efficacy and provide a promising candidate for stem cell-based gene therapy of MS therapy in future.

## Data Availability

All data generated or analyzed data during this study are included in this published article.

## Abbreviations

MS: Multiple sclerosis
CNS: Central nervous system
EAE: Experimental autoimmune encephalomyelitis
IFN-β: Beta interferon
LIF: Leukemia inhibitory factor
MSCs: Mesenchymal stem cells
Tregs: Regulatory T cells
ADSCs: Adipose-derived stem cells
RRMS: Relapsing–remitting multiple sclerosis
PBS: Phosphate buffer saline
SVF: A stromal vascular fraction
GFP: Green fluorescent protein
PFA: Paraformaldehyde
HE: Hematoxylin and eosin
LFB: Luxor Fast Blue
MBP: Myelin Basic Protein
DAPI: Diamidino phenyl indole
IL-17a: Interleukin 17a
TNF α: Tumor necrosis factor α
IFN γ: Interferon γ
